# Genome-Wide DNA Methylation Profiling Reveals Ancestry-Associated Epigenetic Reprogramming in Cervical Intraepithelial Neoplasia

**DOI:** 10.3390/ijms27093986

**Published:** 2026-04-29

**Authors:** Mohamed Masoud, Charu Shastri, Rajarshi Banerjee, Saanvi Dasgupta, Hector Chavarria-Bernal, Karan P. Singh, Jennifer Y. Pierce, Santanu Dasgupta

**Affiliations:** 1 Department of Pathology, Frederick P. Whiddon College of Medicine, University of South Alabama, Mobile, AL 36617, USA; mamasoud@uabmc.edu (M.M.); cshastri@health.southalabama.edu (C.S.); rb2424@jagmail.southalabama.edu (R.B.); hchavarriabernal@health.southalabama.edu (H.C.-B.); 2 Mitchell Cancer Institute, University of South Alabama, Mobile, AL 36604, USA; jypierce@health.southalabama.edu; 3 Heersink School of Medicine, University of Alabama at Birmingham, Birmingham, AL 35294, USA; sdasgupt@uab.edu; 4 Department of Epidemiology and Biostatistics, The University of Texas at Tyler Health Science Center, Tyler, TX 75708, USA; karan.singh@uttyler.edu; 5 Department of Biochemistry and Molecular Biology, Frederick P. Whiddon College of Medicine, University of South Alabama, Mobile, AL 36688, USA; 6 Department of Urology, Frederick P. Whiddon College of Medicine, University of South Alabama, Mobile, AL 36688, USA

**Keywords:** cervical cancer, in situ carcinoma, DNA methylation, ancestral disparity, biomarker, therapeutic target

## Abstract

Cervical cancer (CC) is an alarming global health problem, with predominantly higher incidence, lethal progression, and mortality among women of African ancestry (AA) than women of European ancestry (EA). Although persistent high-risk human papillomavirus (HPV) integration and infection are the key etiological factors, currently available evidence implicates epigenetic reprogramming as a prime contributor to ancestry-associated differences in CC pathogenesis. To address these disparities, we performed genome-wide DNA methylation profiling of HPV-positive cervical intraepithelial neoplasia (CIN) lesions from AA (*n* = 15) and EA (*n* = 15) women. Differential methylation analysis identified a distinct epigenomic landscape in AA-CIN lesions, with widespread hypermethylation and hypomethylation at promoter-associated and regulatory CpG sites. Pathway enrichment analyses highlighted dysregulation of ECM-receptor interaction, focal adhesion, PI3K-Akt, MAPK, Ras, Rap1, and RUNX-dependent transcriptional networks. Comparative analysis across CIN grades (CIN1–CIN3) revealed progressive epigenetic reprogramming affecting cell cycles, cytoskeletal dynamics, signaling, and metabolic pathways. Among hypermethylated tumor suppressor genes, *SH3GL2* and *ARHGAP25* showed significantly higher methylation in AA lesions, accompanied by concomitant loss of their protein expression. MBD1, a methylation-binding regulator, was upregulated in AA-CIN lesions, coinciding with global loss of 5-hydroxymethylcytosine (5hmC), suggesting enhanced transcriptional repression. In contrast, EA lesions retained protein expression and 5hmC levels. Collectively, these findings indicate that early, ancestry-specific epigenetic modifications target tumor suppressor pathways and converge on oncogenic signaling, cytoskeletal remodeling, and cell–cell adhesion. Our study provides mechanistic insight into CC health disparities, identifying *SH3GL2* and *ARHGAP25* hypermethylation as potential biomarkers, and highlighting epigenetic regulation as a contributor to disparate CC progression in AA women.

## 1. Introduction

In 2022, an estimated 662,044 cases and 348,709 deaths worldwide designated cervical cancer (CC) as the fourth leading cause of cancer-associated morbidity and mortality in women [1,2,3]. Despite human papillomavirus (HPV) vaccination-based prevention, screening and early detection, women with African ancestry (AA) are continuously presented with aggressive CC and higher mortality [4,5,6,7,8,9,10]. The death rate is different between EA (2.0 cases per 100,000) and AA women (3.4 cases per 100,000). In addition, AA women have approximately a 1.5 times higher mortality rate due to CC than EA women in Alabama, particularly in the Deep South [5]. These outcomes are evident in the United States and globally among AA women compared to EA women, necessitating the need for a deeper understanding of the biological mechanisms contributing to these inequities. Persistent infection with high-risk human papillomavirus (HPV) is the central driver of CC carcinogenesis; however, HPV infection alone is insufficient to explain the disparities in disease progression and outcomes. Existing and emerging evidence indicates that host genomic and epigenomic factors play critical roles in modulating HPV-driven oncogenesis. Among these, epigenetic alterations, particularly aberrant DNA methylation, have emerged as key regulators of gene expression, chromatin remodeling, and cellular identity during cancer evolution and progression.

DNA methylation may occur early during cellular transformation of the cervical epithelium and accumulate stably, conferring increasing histopathological severity, influencing tumor suppressor gene (TSG) silencing, oncogenic activation, and dysregulation of developmental and differentiation pathways. Genome-wide methylation studies have identified epigenetic reprogramming across cervical intraepithelial neoplasia (CIN) grades, implicating pathways involved in cell adhesion, cytoskeletal remodeling, metabolism, and oncogenic signaling. However, the contribution of ancestry-associated epigenomic differences to early cervical lesions and disease progression remain poorly defined. Methyl-CpG binding proteins, such as methyl-CpG binding domain protein 1 (MBD1), act as key effectors of DNA methylation-mediated transcriptional repression by binding methylated CpG islands and recruiting chromatin-modifying complexes [11,12,13,14,15]. Dysregulation of these proteins can amplify epigenetic silencing of TSGs and promote tumorigenesis. In parallel, global loss of 5-hydroxymethylcytosine (5hmC), a stable epigenetic mark reflecting DNA methylation dynamics, has been associated with malignant transformation and aggressive cancer phenotypes [16,17,18,19].

In this study, we performed comprehensive genome-wide DNA methylation analyses of HPV-positive CIN lesions across progressive histological grades, comparing AA and EA cohorts. We further integrated pathway enrichment, protein–protein interaction network analyses, and validated most frequently methylated genes *SH3GL2* and *ARHGAP25* in matched samples to identify functionally relevant, ancestry-associated epigenetic hallmarks. Our findings reveal that early hypermethylation and silencing of key TSGs, altered methylation machinery, and global epigenetic instability in CIN lesions from AA women may contribute to CC health disparities and identify potential biomarkers for improving early risk stratification and timely interventions.

## 2. Results

### 2.1. Presence of Distinct Epigenomic Landscape in the Early Lesions of Women with African Ancestry

**Figure 1:**
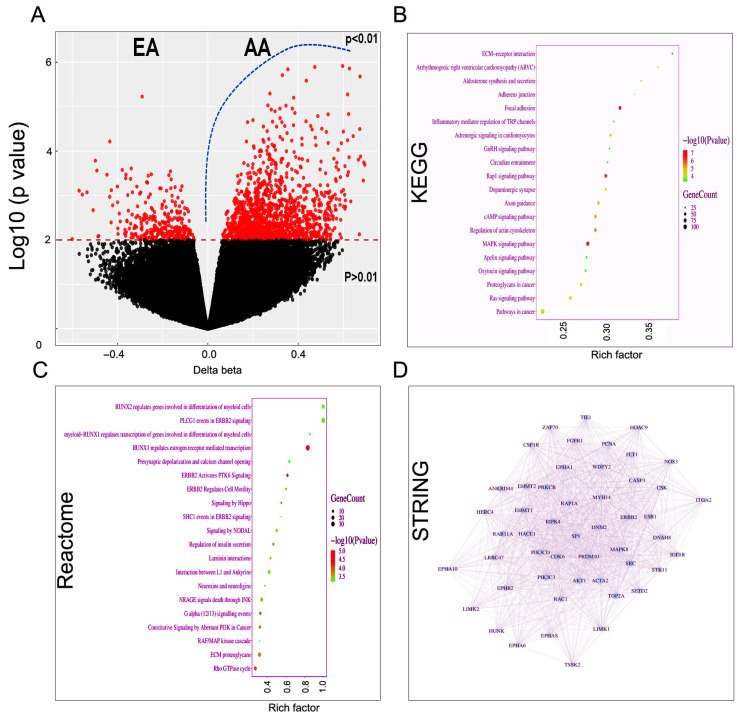
DNA methylation and functional enrichment analysis in cervical intraepithelial neoplasia lesions from women with African and European ancestry. (**A**) Volcano plot showing differential DNA methylation between women with European (EA) and African ancestry (AA). Each point represents an individual CpG site. Red points indicate significantly differentially methylated sites (*p* < 0.01), while black points represent non-significant sites. The *x*-axis shows methylation difference between groups, and the *y*-axis shows log10 (*p* value). (**B**) KEGG pathway enrichment analysis of genes associated with differentially methylated CpG sites. Pathways are ranked by enrichment significance. Dot size represents the number of genes enriched in each pathway, and color indicates log10 (*p* value). The *x*-axis represents the rich factor. (**C**) Bubble plots from reactome analysis depict the top significantly enriched reactome pathways for each gene set. The *x*-axis represents the rich factor (ratio of genes from the input list mapped to a pathway relative to the total number of genes annotated to that pathway), and the *y*-axis lists the enriched reactome pathways. Dot size corresponds to the number of genes (Gene Count) mapped to each pathway, while dot color indicates statistical significance expressed as log10 (*p* value), with warmer colors representing higher significance. (**D**) Protein–protein interaction (PPI) network (STRING) constructed from differentially methylated genes. Nodes represent proteins encoded by differentially methylated genes, and edges represent known or predicted protein–protein interactions. The network highlights highly interconnected genes, suggesting coordinated regulation of key biological pathways.

We examined global methylation changes in HPV-positive CIN lesions obtained from women with AA (*n* = 15) and EA (*n* = 15) ancestry. Global methylation analysis identified a distinct methylation pattern between EA and AA groups, The volcano plot illustrates widespread methylation differences, with a substantial number of CpG sites showing statistically significant differential methylation (*p* < 0.01) (Figure 1A). Both hypermethylated and hypomethylated loci were identified, indicating broad epigenetic reprogramming between the two groups. KEGG pathway analysis further supported these observations, highlighting enrichment in pathways associated with ECM-receptor interaction, adherens junctions, focal adhesion, MAPK signaling, Rap1 signaling, and axon guidance. Several cancer-related pathways were also found to be enriched, indicating that differentially methylated genes may influence signaling networks relevant to cellular communication, migration, and disease-associated processes (Figure 1B). Reactome pathway enrichment analysis revealed significant overrepresentation of pathways related to transcriptional regulation, receptor tyrosine kinase signaling, and cytoskeletal dynamics (Figure 1C). Prominent enrichment of RUNX-dependent transcriptional programs, including RUNX2 and RUNX1-mediated regulatory pathways, indicates involvement of differentiation-associated transcriptional networks. Concurrent enrichment of ERBB2 (a.k.a. HER2) signaling pathways (e.g., PLCG1, PTK6, and SHC1 events), together with the RAF/MAP kinase cascade and aberrant PI3K signaling, supports the activation of canonical mitogenic and oncogenic pathways downstream of receptor tyrosine kinases. Additional enrichment of Hippo and NODAL signaling suggests potential crosstalk between growth control and developmental pathways, while calcium-dependent and G protein-mediated signaling pathways further indicate active intracellular signal transduction. Notably, adhesion and cytoskeleton-related pathways, including laminin interactions, ECM proteoglycans, and the Rho GTPase cycle were also enriched, consistent with the regulation of extracellular matrix organization and cell motility. Collectively, these findings define a coordinated signaling signature integrating transcriptional control, growth factor signaling, and cytoskeletal remodeling. To explore potential functional interactions, a protein–protein interaction (PPI) network was constructed using differentially methylated genes. The resulting network revealed a densely connected structure with multiple highly related hub genes, suggesting coordinated regulation of signaling and adhesion-related pathways. These hub genes may represent key epigenetically regulated drivers underlying the observed biological differences between EA and AA groups (Figure 1D). Collectively, these results indicate that differential DNA methylation is enriched in genes regulating cell adhesion, signaling, and cytoskeletal organization, potentially contributing to ancestry-specific biological phenotypes. Genome-wide methylation analysis of CIN lesions (n = 30) identified a panel of hypermethylated genes predominantly localized to promoter-associated regions (TSS200, TSS1500, 5′UTR), suggesting transcriptional silencing (Table 1). Functional annotation revealed enrichment in pathways related to genomic stability (*MSH2*, *RNF138*), transcriptional regulation (*ZNF292*, *ZNF233*, *ILF2*), cytoskeletal organization (*SEPT8*, *ARHGAP25*), extracellular matrix integrity (*TNXB*, *COL5A3*), and metabolic control (*PCK1*, *THUMPD3*). Notably, several genes demonstrated CpG hypermethylation in promoter regions, consistent with epigenetic repression. The functional clustering indicates coordinated silencing of tumor suppressor pathways during early cervical carcinogenesis.

### 2.2. Distinct Global Methylation Pattern Across Carcinoma In Situ Progression

**Figure 2:**
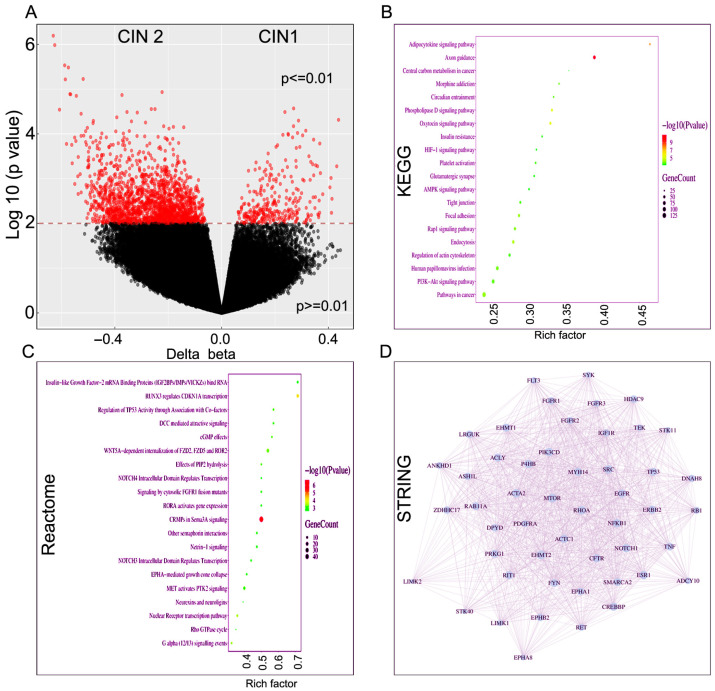
Pattern of DNA methylation and association of enriched functional pathways in grade 1 and grade 2 cervical intraepithelial neoplasia lesions. (**A**) Volcano plot showing differential DNA methylation between grade 1 and grade 2 cervical intraepithelial neoplasia (CIN) lesions. Each dot represents an individual CpG site. Red dots indicate significantly differentially methylated CpG sites (*p* < 0.01), while black dots represent non-significant sites. The *x*-axis shows delta beta (Δβ) values, and the *y*-axis shows log10 (*p* value). The dashed horizontal line indicates the significance threshold. (**B**) KEGG pathway enrichment analysis of genes associated with differentially methylated CpG sites. Dot size corresponds to the number of genes enriched in each pathway (Gene Count), and color represents log10 (*p* value). The *x*-axis indicates the rich factor. (**C**) Reactome enrichment analysis highlighting pathways associated with transcriptional regulation and intracellular signaling cascades. Prominent pathways include regulation of TP53 activity through association with co-factors, RUNX-mediated transcriptional control of CDKN1A, and NOTCH intracellular domain-regulated transcription, indicating modulation of cell cycle checkpoints and stress-response networks. Additionally, significant enrichment of Semaphorin- and netrin-related signaling (e.g., CRMPs in Sema3A signaling, Netrin-1 signaling, and other Semaphorin interactions) suggest involvement of guidance cue pathways that influence cytoskeletal organization and cell migration. The presence of WNT5A-dependent receptor internalization and cGMP-related signaling further indicates activation of non-canonical Wnt and second messenger pathways, collectively reflecting coordinated transcriptional and signaling regulation. (**D**) Protein–protein interaction (PPI) network constructed from differentially methylated genes. Nodes represent proteins encoded by differentially methylated genes, and edges represent known or predicted protein–protein interactions. Highly connected hub genes are centrally positioned within the network, indicating potential regulatory importance.

In addition to comparing the methylation outcomes between AA and EA women, we compared methylation pattern and pathway involvement across the histological grades. First, we compared methylations and associated pathways between CIN1 (*n* = 10) and CIN2 (*n* = 9) lesions. Differential methylation analysis identified widespread CpG methylation differences between CIN1 and CIN2 groups. The volcano plot demonstrates a substantial number of significantly differentially methylated CpG sites (*p* < 0.01), distributed across both hypermethylated and hypomethylated loci (Figure 2A). The distribution pattern indicates broad epigenetic divergence rather than isolated locus-specific changes. KEGG pathway enrichment analysis of genes associated with differentially methylated CpG sites revealed significant enrichment in pathways related to the PI3K-Akt signaling pathway, AMPK signaling pathway, Rap1 signaling pathway, focal adhesion, tight junction, axon guidance, adipocytokine signaling pathway, and pathways in cancer. Several metabolic and signaling pathways were also enriched, including the central carbon metabolism in cancer and HIF-1 signaling. These findings suggest that differential methylation preferentially affects genes involved in cell signaling, cellular adhesion, metabolic regulation, and neuronal guidance mechanisms (Figure 2B). Reactome analysis highlights enrichment in transcriptional regulation and intracellular signaling pathways. Prominent pathways include RUNX3 regulation of CDKN1A transcription, regulation of TP53 activity through association with co-factors, and NOTCH3/NOTCH4 intracellular domain-regulated transcription, indicating regulatory control over cell cycle progression and stress responses. Neuronal signaling pathways such as CRMPs in Sema3A signaling, netrin-1 signaling, and other Semaphorin interactions were also significantly represented, suggesting coordination between transcriptional control and guidance cue signaling. Additionally, enrichment of WNT5A-dependent internalization of FZD2, FZD5 and ROR2 and cGMP effects points to non-canonical Wnt and second messenger signaling involvement (Figure 2C). To explore functional connectivity, a protein–protein interaction (PPI) network was constructed from the differentially methylated genes. The resulting network exhibited dense interconnectivity, with several central hub genes including TP53, EGFR, MTOR, NFKB1, ERBB2, RB1, SRC, and RHOA. These hubs are key regulators of cellular signaling, proliferation, differentiation, and survival, suggesting coordinated epigenetic regulation of major signaling cascades. The high degree of connectivity indicates that methylation alterations may converge on core regulatory networks rather than isolated pathways (Figure 2D). Collectively, these findings demonstrate that differential DNA methylation is enriched in genes governing developmental processes, neuronal differentiation, and major signaling pathways, potentially contributing to ancestry-specific biological phenotypes.

**Figure 3:**
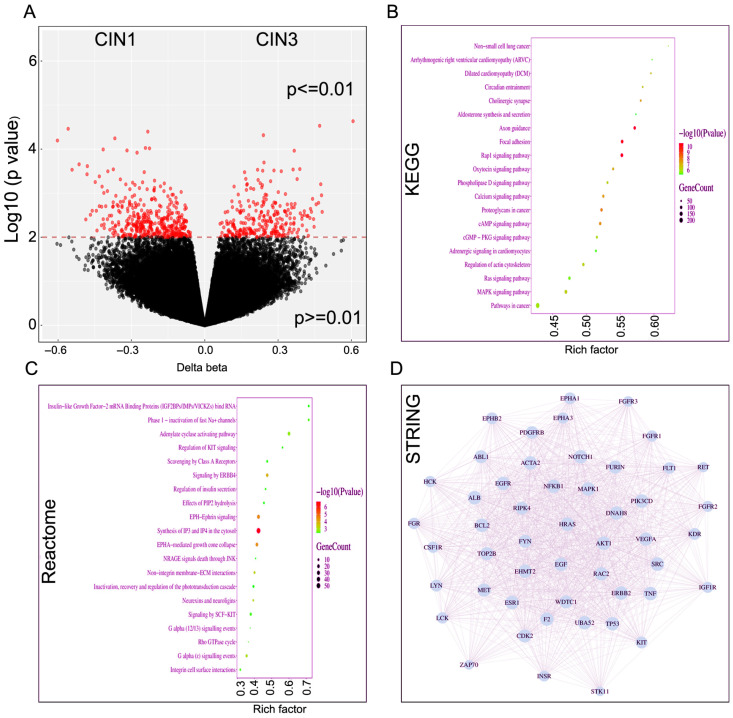
The extent and nature of DNA methylation and involvement of divergent functional pathways in grade 1 and grade 3 cervical intraepithelial neoplasia progression. (**A**) Volcano plot showing differentially expressed genes between grade 1 and grade 3 cervical intraepithelial neoplasia (CIN) lesions. The *x*-axis represents log_2_ fold change (log_2_FC), and the *y*-axis represents log10 (*p* value). Red dots indicate significantly differentially expressed genes (*p* < 0.05), while black dots represent non-significant genes. (**B**) KEGG pathway enrichment analysis of differentially expressed genes. The *y*-axis lists significantly enriched KEGG pathways, and the *x*-axis indicates the rich factor. Dot size reflects the number of genes enriched in each pathway, and color denotes log10 (*p* value). (**C**) Reactome pathways in panel C predominantly involve membrane-associated signal transduction and phosphoinositide metabolism. Key pathways include IGF2BP-mediated mRNA binding, adenylate cyclase–activating signaling, and synthesis of IP3/IP4 in the cytosol, highlighting dynamic regulation of second messenger systems. Enrichment of receptor-driven signaling pathways such as ERBB4 signaling, EPHA-mediated growth cone collapse, SCF–KIT signaling, and integrin cell surface interactions suggests activation of receptor tyrosine kinases and adhesion receptors. The identification of effects of PIP2 hydrolysis and downstream calcium-dependent signaling further support the involvement of phospholipase C-mediated pathways. (**D**) Protein–protein interaction (PPI) network constructed from differentially expressed genes. Nodes represent proteins encoded by DEGs, and edges indicate predicted or validated interactions. Highly connected nodes may represent hub genes involved in key regulatory processes.

We also compared methylation outcomes between CIN1 (*n* = 10) and CIN3 (*n* = 11) lesions. Differential expression analysis identified a substantial number of significantly altered genes between the two experimental conditions (Figure 3A). The volcano plot demonstrates a clear separation of upregulated and downregulated genes based on log_2_ fold change and statistical significance. A large subset of genes met the significance threshold (*p* < 0.05), with numerous transcripts exhibiting strong effect sizes, indicating robust transcriptional reprogramming under experimental conditions. Kyoto Encyclopedia of Genes and Genomes (KEGG) pathway analysis further demonstrated significant enrichment in pathways associated with extracellular matrix (ECM) organization, cell adhesion, signaling cascades, and metabolic processes (Figure 3B). Pathways such as ECM-receptor interaction, focal adhesion, and key signaling pathways displayed high enrichment scores and gene counts, highlighting potential mechanisms underlying the observed phenotypic changes. Reactome analysis demonstrates enrichment in signal transduction and membrane-associated processes, with top pathways including IGF2BP-mediated mRNA binding, adenylate cyclase activating pathway, and effects of PIP2 hydrolysis (Figure 3C). Pathways related to receptor signaling and cellular responsiveness, such as EPH-ephrin signaling, ERBB4 signaling, SCF-KIT signaling, and integrin cell surface interactions are also enriched. Notably, synthesis of IP3 and IP4 in the cytosol show high statistical significance, suggesting active phosphoinositide signaling and calcium-dependent intracellular communication. Protein–protein interaction (PPI) network analysis of the DEGs revealed a highly interconnected network structure (Figure 3D). Several nodes exhibited high connectivity, suggesting potential hub genes that may serve as central regulators in the observed biological processes. The dense interaction patterns indicate coordinated functional relationships among the identified genes. Collectively, these results suggest that the experimental condition induces extensive transcriptional alterations, particularly affecting developmental programs, extracellular matrix remodeling, and key regulatory signaling pathways.

**Figure 4:**
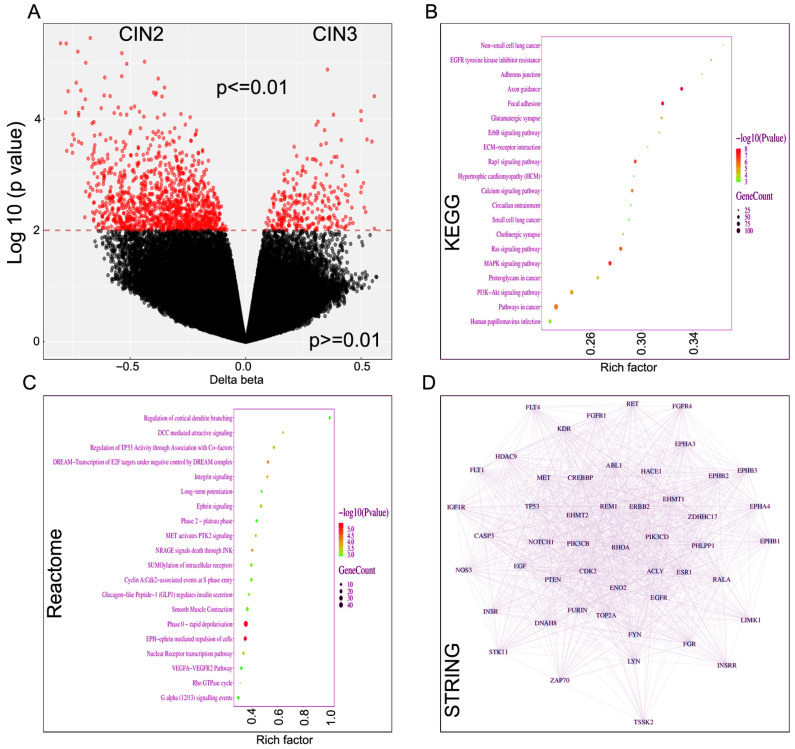
Distribution of DNA methylation and various functional pathway enrichment in CIN1 and CIN3 progression. (**A**) Volcano plot of differentially methylated CpG sites between groups. The *x*-axis represents Δβ (difference in methylation levels), and the *y*-axis shows −log10(*p* value). The red dashed line indicates the significance threshold (*p* < 0.01). Red dots represent significantly differentially methylated sites, while black dots indicate non-significant sites. (**B**) KEGG pathway enrichment analysis of genes associated with significant CpG sites. The *x*-axis shows the rich factor (ratio of differentially methylated genes to total genes in a pathway). Dot size represents gene count, and color indicates enrichment significance (−log10(*p* value)). (**C**) Reactome pathway enrichment analysis demonstrating enrichment of pathways related to cell cycle regulation, adhesion signaling, and cytoskeletal/contractile dynamics. Notable pathways include DREAM-mediated transcriptional repression of E2F targets, cyclin A:CDK2-associated events at S-phase entry, and regulation of TP53 activity, consistent with cell cycle progression control and checkpoint regulation. Concurrent enrichment of integrin signaling, EPH–ephrin-mediated repulsion of cells, Rho GTPase cycling, and SUMOylation of intracellular receptors indicates modulation of cell–cell and cell–matrix interactions coupled with cytoskeletal remodeling. Additional enrichment of smooth muscle contraction, phase 0–rapid depolarization, and long-term potentiation suggests involvement of contractile or electrophysiological regulatory mechanisms. Collectively, these pathways reflect integrated control of proliferation, adhesion, and structural dynamics. (**D**) Protein–protein interaction (PPI) network constructed from significantly enriched genes. Nodes represent proteins encoded by differentially methylated genes, and edges indicate predicted or known protein–protein interactions. Highly connected nodes may represent potential hub genes involved in the biological processes identified.

Next, we compared methylation and affected pathway outcomes in CIN2 (*n* = 9) and CIN3 (*n* = 11) lesions. Genome-wide differential methylation analysis revealed widespread epigenetic alterations between the two groups (Figure 4A). The volcano plot demonstrates the distribution of methylation differences (Δβ values) against statistical significance (log10 *p* value). Using a threshold of *p* < 0.01, numerous CpG sites were identified as significantly differentially methylated. Both hypermethylated (positive Δβ) and hypomethylated (negative Δβ) CpG sites were observed, indicating bidirectional methylation changes across the genome. A subset of CpG sites displayed relatively large effect sizes, suggesting biologically meaningful epigenetic shifts. KEGG pathway enrichment analysis further demonstrated significant enrichment in multiple cancer- and signaling-related pathways (Figure 4B). Notably enriched pathways included PI3K-Akt signaling, MAPK signaling, Ras signaling, focal adhesion, ECM-receptor interaction, ErbB signaling, and pathways in cancer. These pathways are central to cell proliferation, survival, migration, and extracellular matrix interactions, indicating that methylation alterations may influence key regulatory signaling networks. Figure 4C reveals enrichment in cell cycle regulation, receptor signaling, and cytoskeletal or contractile processes. Pathways such as DREAM-mediated transcriptional repression of E2F targets, cyclin A:CDK2-associated events at S-phase entry, and regulation of TP53 activity indicate tight control of proliferation. Concurrent enrichment of EPH-ephrin signaling, integrin signaling, and Rho GTPase cycle suggests coordinated regulation of cell adhesion, migration, and cytoskeletal dynamics. Additionally, pathways related to smooth muscle contraction, phase 0-rapid depolarization, and long-term potentiation indicate broader involvement in excitable or contractile cell functions. Protein–protein interaction (PPI) network analysis revealed a highly interconnected network among genes associated with differentially methylated CpG sites (Figure 4D). Several signaling molecules and receptor tyrosine kinases appeared as central nodes with multiple connections, suggesting potential hub genes that may coordinate downstream pathway effects. The dense interaction pattern supports the notion that epigenetic alterations converge on functionally related signaling modules rather than isolated genes. Overall, these data indicate that differential DNA methylation between the two conditions is enriched in genes regulating development, adhesion, and major oncogenic signaling pathways.

### 2.3. DNA Hypermethylation of SH3GL2 and ARHGAP25 Is an Early Event in CC Health Disparities

**Figure 5:**
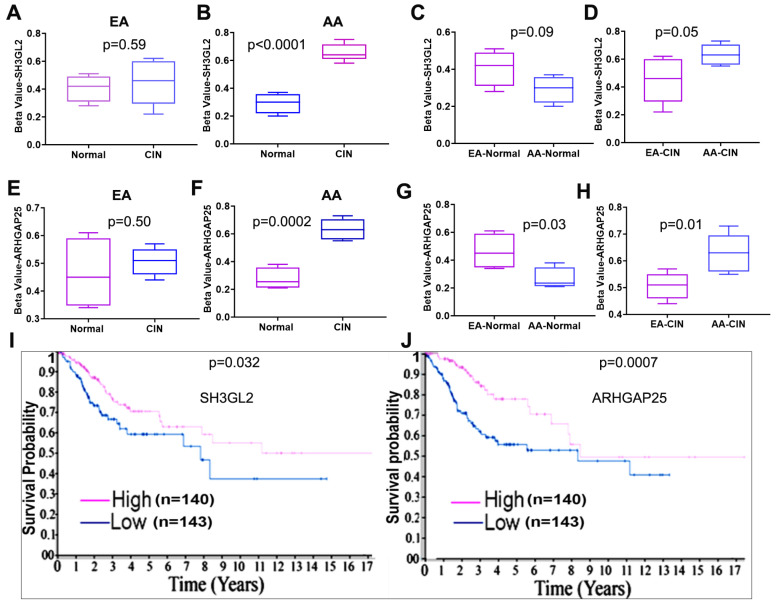
Differential DNA methylation of SH3GL2 and ARHGAP25 by ancestry and association with survival. (**A**,**B**) Comparison of SH3GL2 methylation pattern between normal and CIN samples in EA (**A**) and AA (**B**) cohorts. (**C**) Comparison of SH3GL2 methylation pattern in EA- and AA-normal cohorts. (**D**) Comparison of SH3GL2 methylation pattern between CIN lesions from EA and AA women. (**E**,**F**) Comparison of ARHGAP25 methylation level between normal and CIN samples in EA (**E**) and AA (**F**) cohorts. (**G**) Comparison of ARHGAP25 methylation level in EA- and AA-normal cohorts. (**H**) Comparison of ARHGAP25 methylation pattern between CIN lesions from EA and AA women. (**I**,**J**) The impact of mRNA expression level of SH3GL2 (**I**) and ARHGAP25 (**J**) in predicting survival outcomes in cervical cancer patients. Kaplan–Meier survival analysis was conducted and *p* values were calculated using appropriate statistical methods.

From the panel of top methylated genes, we identified through the genome-wide methylation array (Table 1) that *SH3GL2* and *ARHGAP25* genes appear to be most frequently methylated in CIN lesions from AA women. DNA methylation analyses revealed gene and ancestry-specific differences between normal and CIN samples. For *SH3GL2*, no significant difference (*p* = 0.59) in methylation was observed between normal and CIN lesions in the EA cohort (Figure 5A), whereas a significant increase (*p* < 0.0001) in methylation was detected in CIN compared to normal samples in the AA cohort (Figure 5B). Cross-ancestral comparisons showed no significant difference (*p* = 0.09) in methylation in EA-normal relative to AA-normal tissues (Figure 5C), while CIN lesions from AA women exhibited higher methylation (*p* = 0.05) than EA women (Figure 5D). SH3GL2 can functionally block EGFR expression, a well-known oncogenic driver upregulated in many epithelial malignancies, including CC [20,21,22,23,24]. A similar pattern was observed for *ARHGAP25*. In the EA cohort, methylation levels did not differ significantly (*p* = 0.50) between normal and CIN tissues (Figure 5E), whereas CIN samples in the AA cohort showed significantly elevated (*p* = 0.0002) methylation compared to normal tissues (Figure 5F). When compared across ancestries, EA-normal samples displayed higher methylation (*p* = 0.03) than AA-normal samples (Figure 5G), while AA-CIN samples had a significantly higher (*p* = 0.01) degree of methylation than EA-CIN samples (Figure 5H). ARHGAP25 is a negative regulator of oncogenic Rho GTPases, implicated in actin remodeling, cell polarity, and cell migration in human malignancies, including CC [25,26,27,28,29]. Analysis of The Cancer Genome Atlas (TCGA) data revealed lower mRNA expression of SH3GL2 and ARHGAP5 as worst survival indicators of CC patients (Figure 5I,J).

### 2.4. Early SH3GL2 and ARHGAP25 Methylation Correlated with Loss of Protein Expression in CC Health Disparities

**Figure 6:**
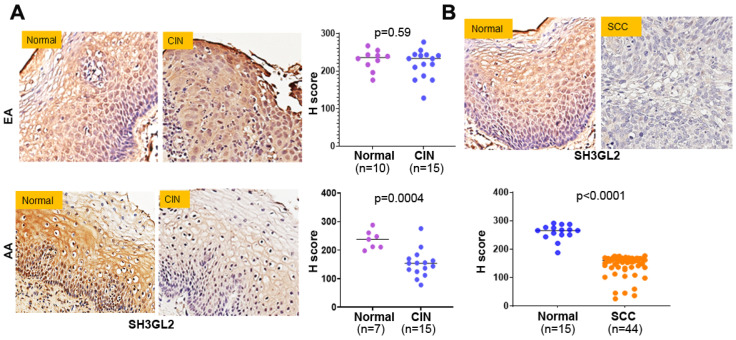
SH3GL2 protein expression in preneoplastic and neoplastic cervical lesions. Cervical intraepithelial neoplasia (**A**) and cervical cancer tissues (**B**) were obtained from women with African and European ancestry. A pathology-guided Aperio digital pathology system was used to determine the cytosolic expression and the score of SH3GL2 in each sample. The dot plots represent the overall staining score in each sample. AA: African ancestry; EA: European ancestry; CIN: cervical intraepithelial neoplasia; SCC: squamous cell carcinoma of the uterine cervix. Magnification ×400.

**Figure 7:**
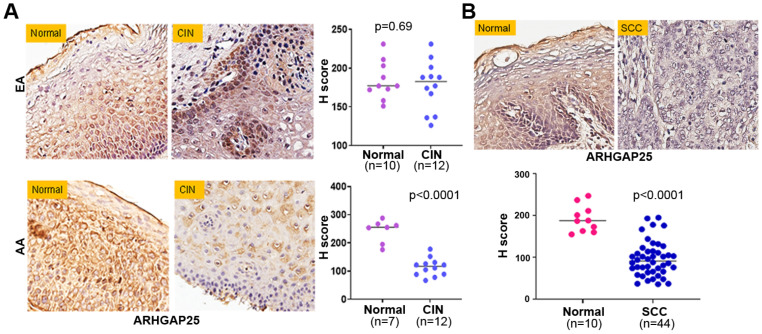
Expression of ARHGAP25 protein in preneoplastic cervical intraepithelial neoplasia and carcinoma tissues. Cervical intraepithelial neoplasia (**A**) and cervical squamous cell carcinoma tissues (**B**) were obtained from women with African and European ancestry. A pathology-guided Aperio digital pathology system was used to determine both membranous and cytosolic expression and the score of ARHGAP25 in individual tissue samples. The dot plots represent the overall staining score in each case. AA: African ancestry; EA: European ancestry; CIN: cervical intraepithelial neoplasia; SCC: squamous cell carcinoma of the uterine cervix. Magnification ×400.

Epigenetic modification of TSGs by increasing the level of DNA methylation is likely to affect its expression at the protein level. To determine the consequences of hypermethylation of SH3GL2 and ARHGAP25, we determined their protein expression by immunohistochemistry in high-grade CIN lesions from both AA and EA groups. These samples include all the cases exhibiting differential degrees of methylation in both EA and AA groups. We did not observe the appreciable loss (*p* = 0.59) of SH3GL2 protein expression in the CIN lesions (*n* = 15) of EA women when compared to controls (*n* = 10) (Figure 6A, upper panel). On the other hand, we confirmed predominant cytoplasmic expression of SH3GL2 proteins and recorded their significant loss (*p* = 0.0004) in the CIN lesions (*n* = 15) of AA women when compared to controls (*n* = 7) (Figure 6A, lower panel). In addition, we recorded the appreciable loss of SH3GL2 protein expression in CC patients (*n* = 44) compared to normal cervical epithelium (*n* = 15) (Figure 6B). While examining the expression pattern of ARHGAP25 in the same samples, we did not observe its appreciable loss (*p* = 0.69) of expression in CIN lesions (*n* = 12) of EA women when compared to controls (*n* = 10) (Figure 7A, upper panel). On the other hand, we confirmed predominant plasma membrane as well as cytoplasmic expression of the ARHGAP25 protein, and observed its significant loss (*p* < 0.0001) of expression in CIN lesions (*n* = 12) of AA women when compared to controls (*n* = 7) (Figure 7A, lower panel). We also confirmed the appreciable loss of the ARHGAP25 protein in CC patients (*n* = 44) compared to normal cervical epithelium (*n* = 10) (Figure 7B).

### 2.5. Abundance of MBD1 Expression in High Grade CIN Lesions from AA Women

**Figure 8:**
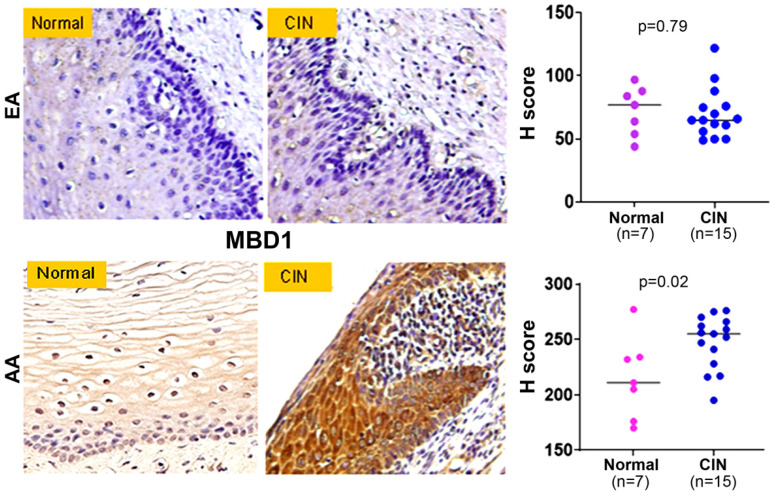
Expression pattern of MBD1 in cervical intraepithelial neoplasia lesions. Formalin-fixed and paraffin-embedded cervical intraepithelial neoplasia lesions obtained from AA and EA women were subjected to immunohistochemistry using MBD1 antibody. A pathology-guided Aperio digital pathology system was used to determine the combined cytosolic and nuclear score of MBD1 in each sample. Magnification ×400.

Methyl-CpG binding domain protein 1 (MBD1) is a central regulator of TSGs, which binds to methylated CpG islands and couples DNA methylation to transcriptional repression, leading to silencing of TSGs [11,12,13,14,15]. Silencing of MBD1 reversed therapy resistance in pancreatic cancer [11] and its upregulation promoted pancreatic tumorigenesis [12]. We measured MBD1 expression by IHC using a pre-optimized antibody. We confirmed predominant nuclear as well as cytoplasmic expression of the MBD1 protein and observed its significant abundance (*p* = 0.02) of expression (Figure 8, lower panel) in the CIN lesions (*n* = 15) of AA women when compared to controls (*n* = 7). However, we did not observe an appreciable difference (*p* = 0.69) of the MBD1 protein expression (Figure 8, upper panel) in the CIN lesions (*n* = 15) of EA women when compared to controls (*n* = 7).

### 2.6. Predominant Loss of 5hmC Methylation Mark in CIN Lesion from AA Women with MBD1 Upregulation

**Figure 9:**
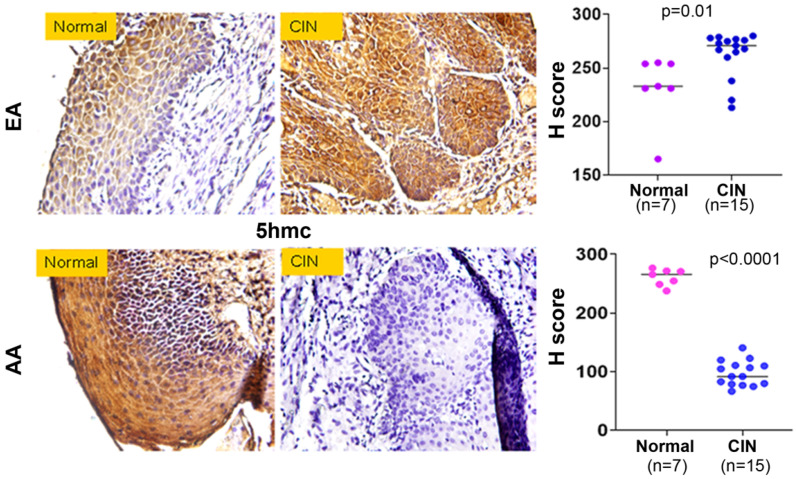
Expression pattern of 5-hydroxymethylcytosine in preneoplastic cervical epithelial lesions. Formalin-fixed paraffin and embedded cervical intraepithelial neoplasia lesions were obtained from AA and EA women undergoing cervical cancer screening. The sections (5 µm) were then stained with anti-human 5-hydroxymethylcytosine antibody for immunohistochemical detection. An Aperio digital pathology system was utilized to determine the combined cytosolic and nuclear score in each section. Magnification ×400.

A well-studied epigenetic change includes an addition of a methyl group on the 5-position of the cytosine (5mC) base in a CpG dinucleotide, which eventually oxidized into 5 hydroxymethylcytosine (5hmC), a more stable mark of the methylation state [16,17,18,19,30]. Accumulation of these methylation marks in CpG-rich regions around the transcriptional start site (TSS) of genes leads to chromatin organization, resulting in alterations of locus-specific transcriptional activity. The 5hmC is regarded as a marker for global DNA methylation. We conducted IHC analysis on the same cohorts of samples described above. We confirmed predominant nuclear as well as cytoplasmic expression of 5hmC expression and observed its significant loss (*p* < 0.0001) of expression (Figure 9, lower panel) in the CIN lesions (*n* = 15) of AA women when compared to controls (*n* = 7). Surprisingly, we observed a markedly higher expression of (*p* = 0.01) of 5hmC (Figure 9, upper panel) in the CIN lesions (*n* = 15) of EA women when compared to controls (*n* = 7).

## 3. Discussion

Despite remarkable improvement in CC screening and prevention strategies, AA women continue to experience a higher rate of mortality from aggressive disease burden [7,31,32,33]. Thus, a deeper understanding of the biological basis of this disparity is warranted. Silencing of TSGs through DNA hypermethylation, inactivating mutations, and allelic loss are divergent mechanisms and essential events driving cancer initiation and progression. In addition, TSG inactivation may occur through various other mechanisms including miRNA or microbial interplay with the human genome as evident for CC, which is primarily driven by HPV carcinogenesis. HPV integration could potentially result in the inactivation of key gatekeeper genes associated with the prevention of tumorigenic development through the hypermethylation of nuclear genomic DNA of the host epithelium. Interestingly, the incidental rate and highly aggressive biological progression recorded in AA women with CC reflects the involvement of distinct molecular pathways. These divergent pathways could be associated with increased susceptibility towards HPV integration and infection and driven lethality. Identification and characterization of the key TSGs in various ancestral populations at the earliest timepoint, such as preneoplastic continuum, would be ideal for disease prevention or delaying disease onset.

In this study, we identified a distinct epigenomic landscape in early CIN lesions of AA women as opposed to EA women, characterized by widespread differential DNA methylation affecting key oncogenic and tumor-suppressive pathways. Genome-wide methylation profiling revealed enrichment of signaling networks central to CC carcinogenesis, including PI3K-Akt, MAPK, Rap1, ErbB, focal adhesion, ECM-receptor interaction, NOTCH and axon guidance pathways [34,35,36,37,38,39,40,41,42,43,44,45,46,47,48]. Reactome pathway enrichment analysis further highlighted dysregulation of RUNX-dependent transcriptional programs, ERBB2 signaling, Hippo signaling, Rho GTPase cycling, and TP53 regulatory networks [49,50,51,52,53,54,55,56,57,58,59,60,61,62,63,64,65]. These pathways converge on core biological processes governing proliferation, differentiation, adhesion, cytoskeletal remodeling, and survival mechanisms known to be hijacked during HPV-driven transformation. Importantly, the dense protein–protein interaction (PPI) networks and identification of hub regulators such as TP53, EGFR, MTOR, ERBB2, RB1, SRC, and RHOA [43,52,54,60,61,62,63,64,65,66,67,68,69,70,71,72,73,74,75,76,77,78,79,80] suggest that methylation changes are not discrete but rather converge on coordinated oncogenic signaling modules.

Analyses across progressive histological grades (CIN1–CIN3) showed that epigenetic divergence increases with lesion severity and preferentially targets genes involved in cell cycle control, developmental signaling, and metabolic reprogramming. Enrichment of DREAM complex-mediated E2F repression, cyclin A/CDK2 signaling, TP53 regulation, and non-canonical Wnt signaling indicates that methylation remodeling accompanies cell cycle rewiring and stress adaptation during malignant progression [20,61,62,63,64,65,66,81,82,83,84,85,86,87,88,89,90,91,92]. Concurrent enrichment of ECM organization, integrin signaling, and phosphoinositide/calcium signaling pathways further supports a model in which early epigenetic alterations promote microenvironmental adaptation and cytoskeletal plasticity; hallmarks of tumor invasion. Together, these findings suggest that epigenetic rewiring is an early and progressive event in CC carcinogenesis.

The present analysis demonstrates ancestry-specific differences in DNA methylation patterns of *SH3GL2* and *ARHGAP25*, particularly during the transition from normal to CIN. Notably, significant hypermethylation of both genes was observed in CIN samples from the AA cohort but not in the EA cohort, suggesting that epigenetic alterations associated with disease progression may be more pronounced in AA individuals. In contrast, EA samples exhibited relatively higher baseline methylation in normal tissues, particularly for *ARHGAP25*, indicating that differences between ancestries may lie not only in absolute methylation levels but also in the nature and type of epigenetic change during neoplastic development. These findings point to potentially distinct biological pathways or regulatory mechanisms underlying CIN progression across divergent populations. SH3GL2 negatively regulates EGFR signaling, and its promoter is hypermethylated in epithelial malignancies [21,22,23,24]. Loss of SH3GL2 may trigger EGFR-driven proliferation and MAPK/PI3K pathway activation, which are prominently enriched in our dataset. Similarly, ARHGAP25 functions as a negative regulator of Rho GTPases, which govern actin remodeling, cell migration and polarity [25,26,27,28,29]. Its hypermethylation and concomitant loss of protein expression suggest derepression of Rho-mediated cytoskeletal dynamics. This is also consistent with the observed enrichment of Rho GTPase and adhesion-related pathways. Importantly, the survival analyses indicate that higher methylation levels are associated with improved clinical outcomes, suggesting a potential protective or prognostic role for methylation at these loci. While promoter hypermethylation is often linked to gene silencing, the observed association with better survival raises the possibility that methylation of *SH3GL2* and *ARHGAP25* may suppress pathways involved in tumor progression or reflect less aggressive disease phenotypes. Alternatively, these methylation patterns may serve as surrogate biomarkers of broader epigenetic states linked to favorable prognosis. Collectively, these results underscore the importance of incorporating population-specific epigenetic data into cancer research and highlighting the potential utility of *SH3GL2* and *ARHGAP25* methylation as prognostic biomarkers. Further studies are warranted to elucidate the functional consequences of these methylation changes and their role in contributing to observed disparities in disease outcomes.

The detection of promoter-associated CpG hypermethylation (TSS200, TSS1500, 5′UTR) predominantly among differentially methylated loci supports transcriptional repression of TSGs. Promoter hypermethylation involving one or both alleles are mechanisms of gene silencing in cancer and frequently occur during neoplastic transformation. Our data suggest that coordinated epigenetic silencing of TSGs involved in maintaining genomic stability, cytoskeletal regulation, and extracellular matrix organization may predispose AA women to exhibit more aggressive biological behavior. Importantly, methylation-associated gene silencing was validated at the protein level. High-grade AA-CIN lesions showed significant loss of SH3GL2 and ARHGAP25 expression, whereas lesions from EA women did not exhibit comparable reductions. This ancestry-specific epigenetic repression suggests that differential DNA methylation may contribute to distinct biological behavior of early lesions. The coordinated upregulation of MBD1, a methyl-CpG binding protein linking DNA methylation to transcriptional repression, provides a potential mechanistic explanation. Elevated MBD1 expression in AA-CIN lesions may reinforce transcriptional silencing of targeted or selective TSGs by stabilizing methylated chromatin states, thereby amplifying epigenetic repression.

Further supporting our notion, we observed a predominant loss of the 5hmC mark in AA-CIN lesions, whereas EA lesions displayed a relative preservation or increase of 5hmC. Because 5hmC is associated with active chromatin and epigenetic plasticity, its depletion suggests a potential shift toward a more transcriptionally repressive and stable methylation landscape. The inverse relationship between MBD1 upregulation and 5hmC loss in AA lesions supports the hypothesis that early cervical neoplasia in this population is characterized by reinforced epigenetic silencing. Collectively, these findings indicate that ancestry-associated differences in DNA methylation, methylation-binding protein abundance, and hydroxymethylation status may contribute to differential TSG regulation during early cervical carcinogenesis.

Due to the small sample size within individual CIN strata, we employed a complementary strategy combining (i) ancestry-based analysis across the full CIN cohort and (ii) CIN progression analysis independent of ancestry. This approach improved statistical power and enabled identification of both ancestry and stage-associated epigenetic alterations. Importantly, ancestry-associated differences aligned with progressive methylation changes across CIN1–CIN3, indicating that these signatures are embedded within higher-risk epigenetic states rather than confined to a single stage. Consistent involvement of pathways such as PI3K-Akt, MAPK, and ECM-receptor interaction further supports biological coherence. However, larger, stage-stratified studies will be useful to validate ancestry-specific effects within individual CIN grades. The small sample size also limits the detection of subtle changes, with a reliance on statistical significance without Δβ thresholds. Moreover, potential batch effects could have occurred due to non-randomized processing and lack of formal correction. Although this study performed methylation analysis of CIN lesions rather than invasive CCs, the findings have implications for disease progression and risk stratification. CIN2/3 lesions are heterogeneous, with only a subset progressing to cancer. We observed widespread differential methylation in AA lesions, including enrichment of PI3K-Akt, MAPK, focal adhesion, and ECM-related pathways, suggesting an epigenetic landscape permissive of persistence and progression. Hypermethylation-mediated silencing of TSGs (e.g., *SH3GL2*, *ARHGAP25*), along with reduced 5hmC and increased MBD1 expression, indicate enhanced transcriptional repression and potentially higher biological risk. Integration of these epigenetic features with clinical parameters may improve predictive models of CIN progression. Of note, loss of the key altered genes *SH3GL2* and *ARHGAP25* was validated at protein level in CIN lesions as well as cervical carcinoma tissues, reflecting the translational relevance and potential usefulness of our findings.

In conclusion, CIN lesions from AA women exhibit a distinct, more repressive epigenetic profile characterized by promoter hypermethylation of TSGs, activation of oncogenic pathways, increased MBD1 expression, and global 5hmC loss. These changes converge on pathways regulating proliferation, adhesion, and cytoskeletal dynamics and may contribute to cancer health disparities. Future work should include larger, longitudinal, and stage-stratified cohorts, functional validation of key genes such as *SH3GL2* and *ARHGAP25*, and integration of methylation with transcriptomic and chromatin-level data. Incorporating environmental exposures, viral characteristics and subtypes, and host genetic variation will further clarify drivers of epigenetic remodeling. Longitudinal studies tracking CIN lesions, including early invasive cases, will be important to assess whether these methylation signatures are associated with lesion persistence, increased likelihood of progression, and clinical outcomes, and to evaluate their potential utility as prognostic biomarkers.

## 4. Materials and Methods

### 4.1. Patient Cohort and Ethical Statement

Formalin-fixed paraffin-embedded (FFPE) biopsied tissues from women with a confirmed histopathological diagnosis of various grades of cervical intraepithelial neoplasia (CIN1-3, *n* = 30) were collected (Table 2). Of the total thirty cases, fifteen women had African ancestry (AA, self-reported) and fifteen women had European ancestry (EA), and all were positive for human papillomavirus (HPV) infection. Of these cases, ten were CIN1, nine were CIN2, and eleven were CIN3 lesions. All biospecimens, including an additional cohort of 44 cervical cancer patients (FFPE), were collected from the de-identified subject under an IRB-approved protocol from the University of South Alabama (#20–222). Of the SCC cases, 24 were from stage I and 20 were from stage III. This study was approved by the Ethics Committee of Medicine, University of South Alabama. Informed consent was obtained from all the de-identified subjects, and only relevant clinical information such as age, grade, diagnosis, HPV status, ancestry, etc., was collected for statistical comparison. All methods were performed following the relevant guidelines and regulations. Due to the limited availability of well-characterized FFPE CIN specimens stratified by ancestry, a formal a priori power calculation was constrained. However, with 15 samples per group (AA vs. EA; α = 0.05, two-sided), the study is estimated to have adequate (~80%) power to detect large effect sizes (Cohen’s d ≥ 0.9).

### 4.2. Isolation and Purification of DNA Samples

High-quality genomic DNA was isolated from micro-dissected FPPE samples containing at least 80% cervical epithelial tissues using a QIAamp DNA FFPE Advanced Kit (#56604, Qiagen, Germantown, MD, USA). Eight to ten sections (10 µm) were cut to obtain a sufficient amount of DNA samples. DNA concentration was measured using the QIAxpert system (#9002340, Qiagen). The genomic DNA was subjected to quality control and only samples that passed QC were used for the downstream analysis.

### 4.3. EPIC BeadChip Methylation Array, Raw Data Pre-Processing, and Normalization

The DNA samples were then subjected to bisulfite sequencing on the Illumina Infinium MethylationEPIC array BeadChip (850K) platform. The 850K BeadChip array allows quantitative evaluation of over 850,000 methylation sites across the genome at the single nucleotide level on multiple sample types, including FFPE samples. Bisulfite conversion was controlled by qPCR. For quality control, one assay targeting a methylated region of DNAJC15 and two assays targeting the GNAS locus (one assay for the unmethylated allele and one assay for the methylated allele) were utilized. Blood-derived deaminated DNA was amplified in parallel, which served as the positive control. A sample passed the quality control when the received ct-value of the two GNAS loci or the DNAJC15 locus reached the threshold no later than five cycles compared to the positive control. EWAS protocol was performed in accordance with Illumina’s protocol. Comparative analysis of the data was performed with the Biocionductor R package (v3.19) Chip Analysis Methylation Pipeline (ChAMP). The ChAMP filter allows removal of probes with detection of *p* values > 0.01, low bead counts, non-CpG probes, probes overlapping known SNPs, multi-hit probes, and probes located on sex chromosomes. Data were then normalized with BMIQ.

### 4.4. Identification of Differentially Methylated Probes and Regions and Quality Control

Differentially methylated probes (DMPs) were identified using a Benjamini–Hochberg adjusted *p* value < 0.05 with no threshold set on the methylation differences. On the other hand, differentially methylated regions (DMRs) were identified as regions displaying a global methylation difference between the two groups. DMRs were identified using the Bumphunter method, considering the maximum distance between two consecutive probes to be 300 bp. The reference genome GRCh37/hg19 was used as the reference genome and obtained from the UCSC genome browser. Quality control of the Illumina Infinium EPIC array was performed across all samples; none exceeded the failed probe threshold (>0.1), so all were retained. Sample distances were calculated using filtered probes prior to normalization. Differentially methylated probes were identified using a Benjamini–Hochberg adjusted *p* value < 0.05 without a Δβ cutoff to maximize sensitivity, retaining small effect sizes. Samples followed standard Illumina protocols, but no explicit randomization or batch correction was applied. All analyses were performed exclusively on HPV-positive cervical intraepithelial neoplasia (CIN) samples, and no normal cervical epithelial controls were included. Therefore, methylation differences represent ancestry-associated variation within the disease context rather than case–control differences between unmatched healthy and diseased tissue. Top altered genes were validated at protein level using appropriate normal cervical, CIN lesions, and carcinoma tissues.

### 4.5. Gene Ontology and KEGG Function Enrichment Analysis

The function of the gene was described by gene ontology (GO) function enrichment analysis of the gene where the differential methylation level site was located (combined with the results of GO annotation). The Kyoto Encyclopedia of Genes and Genomes (KEGG) pathway analysis was conducted on the genes based on the location of the differential methylation level sites (combined with the results of KEGG annotation). The number of genes included in each KEGG pathway was counted and the significance of gene enrichment in each pathway was calculated using the hypergeometric distribution test method. The calculated result returns a *p* value describing the significance of enrichment, with a small *p* value (≤0.05) indicating that the gene is enriched in the pathway. The enrichment score was calculated in the same way as that for GO analysis. Pathway analysis of the differentially methylated genes was used to find pathways that enrich these genes. These combinatorial approaches determined the differentially methylated genes related to the corresponding changes in various cellular pathways.

### 4.6. Disease Annotation Function and Reactome Metabolic Pathway Analysis

The disease annotation function analysis was utilized to perform the function annotation and classification of the disease types in the DisGeNET disease database for these differentially methylated genes. Reactome metabolic pathway analysis was used to annotate and classify the metabolic pathways in the Reactome database for these genes as well.

### 4.7. Promoter Methylation Analysis

Bisulfite-modified DNA was used as a template for fluorescence-based real-time PCR. Amplification was carried out in 20 μL (triplicate) containing 3 μL of bisulfite-modified DNA; 600 nmol/L of forward and reverse primers; 200 nmol/L probe; 5U of platinum Taq polymerase; 200 μmol/L each of dATP, dCTP, and dGTP; 200 μmol/L dTTP, and 5.5 mmol/L MgCl_2_. Primers and probes were designed to specifically amplify the promoters of SH3GL2 and ARAHGAP25 and a reference gene, *ACTB*. Amplifications were carried out as: 95 °C for 3 min, followed by 50 cycles at 95 °C for 15 s, and then 60 °C to 62 °C for 1 min. Amplifications were carried out in 384-well plates in a 7900-sequence detector (Perkin-Elmer Applied Biosystems, Waltham, MA, USA) and were analyzed by a sequence detector system (SDS 2.2.1; Applied Biosystems). Each plate included patient DNA samples, positive (in vitro methylated leukocyte DNA) and negative (normal leukocyte DNA or DNA from a known unmethylated cell line) controls, and multiple water blanks. Leukocyte DNA from a healthy individual was methylated in vitro with excess SssI methyltransferase (New England Biolabs Inc., Ipswich, MA, USA) to generate completely methylated DNA, and serial dilutions (90–0.009 ng) of this DNA were used to construct a calibration curve for each plate. The relative level of methylated DNA for each gene in each sample was determined as a ratio of MSP-amplified gene to *ACTB* (reference gene) and then multiplied by 1000 for easier tabulation (average value of triplicates of gene of interest divided by the average value of triplicates of *ACTB* × 1000) [92,93,94,95]. The samples were categorized as unmethylated or methylated on the basis of the assay sensitivity.

### 4.8. Immunohistochemistry

Immunohistochemistry (IHC) was performed on a 5 µm FFPE section of cervical intraepithelial neoplasia (CIN) lesions. HPV-negative cervical lesions were used as controls. Pre-validated primary antibodies against SH3GL2 (#NBP2-57228) and ARHGAP25 (#NBP2-03024) (Novus Biologicals), as well as 5-hydroxymethylcytosine (5hmC) and MBD1 (#A-1018 and #A-1006; Epigentek, Farmingdale, NY, USA), were used for IHC analysis. IHC staining was evaluated using the Aperio digital pathology system (Leica Biosciences, Buffalo Grove, IL, USA) under the guidance of a pathologist (CS) [96,97,98]. The system quantitatively assesses membranous, cytoplasmic, and nuclear protein expression using a three-tier intensity scale (+1, +2, and +3) combined with the percentage of positive cells (0–100%). For each subject, the entire tissue section was analyzed to determine staining intensity and the proportion of positive cells. Cytoplasmic, nuclear, and membranous scores generated by the digital pathology system were used as indices for comparative analyses between AA and EA groups.

### 4.9. Transcriptomic Profiling and Statistical Analysis

Transcriptomic signature-based survival predictions for SH3GL2 and ARHGAP25 were obtained from the Human Protein Atlas database [99]. Statistical significance was assessed using Student’s *t*-tests, chi-squared tests, Fisher’s exact tests, or one-way ANOVA, as appropriate. A *p* value < 0.05 was considered statistically significant. All statistical tests were two-sided, with 95% confidence intervals. GraphPad Prism software (v 10) was used for statistical analyses of the IHC datasets.

## 5. Conclusions

In summary, our findings reveal a distinct early epigenomic landscape in CIN lesions from women of African ancestry, characterized by promoter hypermethylation of tumor suppressor genes, upregulation of MBD1, loss of 5hmC, and coordinated remodeling of adhesion- and signaling-related pathways. Hypermethylation and silencing of *SH3GL2* and *ARHGAP25* emerge as early events associated with adverse molecular features and worst survival. These results suggest that epigenetic reprogramming contributes to cervical cancer health disparities and highlight potential biomarkers and therapeutic targets for precision prevention strategies.

## Figures and Tables

**Table 1:** Top methylated genes in the CIN lesions.

Gene Name	Gene Feature	Chromosomal Location	Function
*THUMPD3*	TSS200, cg19372952	3p25	tRNA methylation
*ZNF292*	Body, cg26565472	6q14	Transcriptional regulation
*SERPINA1*	TSS1500, cg00108569	14q32	Serine protease inhibitor
*ILF2*	TSS200, cg24951781	1q21	Transcriptional regulation
*SEPT8*	TSS1500, cg01528321	5q31	Cytoskeletal organization
*TSPAN14*	5′UTR	10q23	Regulation of Notch signaling
*NPTX1*	3′UTR, cg13182916	17q25	Synapse remodeling
*TNXB*	Body cg22143115	6p21	ECM cohesion
*PKIB*	TSS1500, cg12077487	6q23	Protein kinase inhibitor
*BRUNOL5*	Body cg16551414	19p13	Nucleotide binding
*CYYR1*	TSS1500, cg02896251	21q21	Integral component of membrane
*HBEGF*	3′UTR cg23352621	5q31	Enables growth factor activity
*HIVEP3*	5′UTR	1p34	Transcriptional regulation
*MSH2*	Body cg16131972	2p21	Mismatch repair
*SLC13A3*	5′UTR cg08314795	20q13	High-affinity sodium-dicarboxylate cotransporter
*COL5A3*	Body cg09293560	19p13	Type V collagen
*REPIN1*	TSS200 cg16994880	7q36	Fatty acid transport
*PCK1*	3′UTR cg17854747	20q13	Regulate gluconeogenesis
*RNF138*	5′UTR cg20291363	18q12	DNA damage response
*NKAIN1*	TSS1500, cg12497171	1p35	Interacts with the beta subunit of Na, K-ATPase
*ZNF233*	TSS1500	19q13	Transcriptional regulation
*SH3GL2*	TSS1500	9p22	Synaptic vesicle endocytosis
*ARHGAP25*	TSS1500	2p13	Negative regulators of Rho GTPases

**Table 2:** Demographics of the cervical intraepithelial lesions.

Patient ID	Age	Grade ^1^	Ancestry ^2^	HPV ^3^	Marital Status ^4^	BMI ^5^	Birth Control	FHC ^6^	SMO/ DRK ^7^	No. of Pregnancies	No. of Births	Age of Menarche
HC1	24	CIN1	AA	+	UM	27	Y	N	Y/Y	1	1	NR
HC5	25	CIN1	AA	+	M	NR	NR	N	N/UK	1	0	12
HC7	39	CIN1	AA	+	UM	28	Y	Y	N/N	6	6	NR
HC8	36	CIN1	AA	+	S	34	NR	N	N/Y	3	3	NR
HC12	26	CIN1	EA	+	UM	29	Y	N	N/N	3	3	13
HC13	38	CIN1	EA	+	DIV	32	Y	Y	Y/Y	4	2	14
HC15	50	CIN1	EA	+	DIV	25	NR	Y	N/Y	6	4	NR
HC23	58	CIN1	EA	+	M	56	Y	Y	N/N	2	2	13
HC24	37	CIN1	EA	+	S	46	N	N	N/N	8	7	15
HC26	24	CIN1	EA	+	UM	45	Y	Y	N/N	4	3	11
HC3	49	CIN2	AA	+	UM	30	Y	N	N/N	0	0	NR
HC6	27	CIN2	AA	+	M	52	NR	Y	Y/Y	1	1	NR
HC10	50	CIN2	AA	+	UM	31	Y	N	N/N	0	0	NR
HC11	33	CIN2	AA	+	M	41	Y	Y	N/Y	1	1	12
HC14	39	CIN2	EA	+	UM	23	NR	Y	Y/Y	2	1	NR
HC20	31	CIN2	EA	+	M	33	NR	N	N/Y	5	1	NR
HC25	25	CIN2	EA	+	S	34	Y	Y	N/N	0	0	NR
HC27	33	CIN2	EA	+	M	26	NR	Y	Y/Y	0	0	NR
HC28	46	CIN2	EA	+	UM	20	Y	Y	N/Y	3	3	NR
HC18	28	CIN3	AA	+	UM	33	Y	Y	Y/Y	6	3	NR
HC2	44	CIN3	AA	+	S	25	NR	N	Y/N	0	0	NR
HC4	48	CIN3	AA	+	DIV	32	NR	Y	Y/Y	1	1	NR
HC9	29	CIN3	AA	+	NR	24	N	N	N/Y	1	1	NR
HC16	40	CIN3	AA	+	S	22	N	Y	N/N	2	2	NR
HC17	25	CIN3	AA	+	S	29	Y	N	N/N	0	0	NR
HC29	26	CIN3	AA	+	M	32	Y	NR	N/N	4	3	NR
HC30	63	CIN3	AA	+	DIV	33	NR	Y	N/Y	2	2	15
HC19	43	CIN3	EA	+	M	22	NR	N	N/Y	0	0	NR
HC21	48	CIN3	EA	+	UM	29	NR	N	N/N	0	0	NR
HC22	35	CIN3	EA	+	S	42	YN	N	N/Y	6	5	13

^1^ CIN: cervical intraepithelial neoplasia; ^2^ AA: African ancestry; EA: European ancestry; ^3^ HPV: human papillomavirus. ^4^ Marital status: UM: Unmarried; M: Married; S: Single; DIV: Divorced. ^5^ BMI: Body mass index; ^6^ FHC: Family history of cancer; ^7^ SMO/DRK: Smoking and drinking; NR: Not reported; Y: Yes; N: No.

## Data Availability

All raw data files of methylation analyses have been submitted to the Gene Expression Omnibus database (GEO #GSE327663). Transcriptomes and survival analysis data are available in Human Protein Atlas data base. Other data are available upon request.

## Abbreviations

The following abbreviations are used in this manuscript:

CC	Cervical cancer
CIN	Cervical intraepithelial neoplasia
HPV	Human papillomavirus
ECM	Extracellular matrix
5hmC	5 hydroxymethylcytosine
TSS	Transcription start site
CpG	5’-C-phosphate-G-3’
KEGG	Kyoto Encyclopedia of Genes and Genomes
GO	Gene ontology
PPI	Protein–protein interaction
IHC	Immunohistochemistry
FFPE	Formalin-fixed paraffin-embedded
DEGs	Differential expression of genes
GEO	Gene Expression Omnibus.
